# (2*Z*)-Ethyl 5-(4-methoxy­phen­yl)-7-methyl-3-oxo-2-(3,4,5-trimethoxy­benzyl­idene)-3,5-dihydro-2*H*-thia­zolo[3,2-*a*]pyrimidine-6-carboxyl­ate

**DOI:** 10.1107/S1600536808041019

**Published:** 2009-01-08

**Authors:** Zhao-Hui Hou

**Affiliations:** a Department of Chemistry and Chemical Engineering, Hunan Institute of Science and Technology, Yueyang 414000, People’s Republic of China

## Abstract

In the title compound, C_27_H_28_N_2_O_7_S, the dihedral angles between the thia­zole ring and the mono- and tris­ubstituted benzene rings are 87.8 (2) and 17.9 (3)°, respectively. The dihydro­pyrimidine ring adopts a flattened boat conformation. In the crystal structure, π–π stacking occurs [centroid–centroid separation = 3.6611 (11) Å].

## Related literature

For background to the biological properties of fused pyrimidine derivatives, see: Ashok *et al.* (2007 ▶); Monks *et al.* (1991 ▶). For related structures, see: Liu *et al.* (2004*a* ▶,*b* ▶); Sridhar *et al.* (2006 ▶).
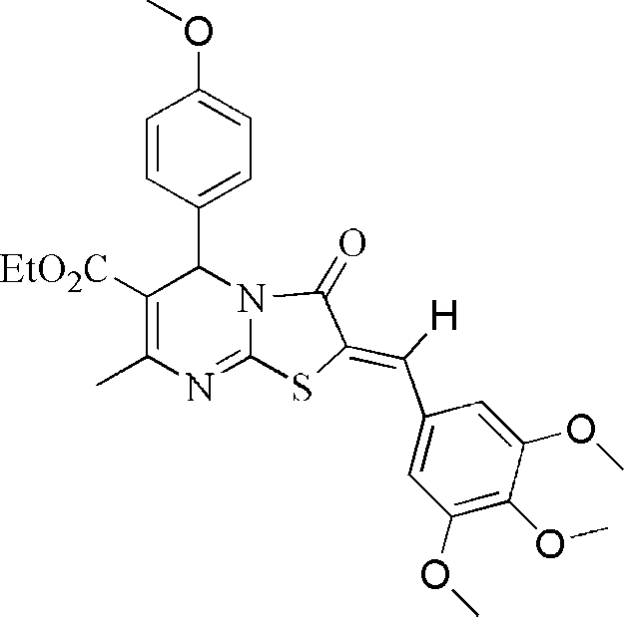


## Experimental

### 

#### Crystal data

C_27_H_28_N_2_O_7_S
*M* *_r_* = 524.57
Triclinic, 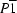

*a* = 10.485 (2) Å
*b* = 10.854 (2) Å
*c* = 11.318 (2) Å
α = 83.42 (3)°
β = 77.65 (3)°
γ = 89.00 (3)°
*V* = 1250.0 (4) Å^3^
*Z* = 2
Mo *K*α radiation
μ = 0.18 mm^−1^
*T* = 113 (2) K
0.24 × 0.18 × 0.16 mm

#### Data collection

Rigaku Saturn CCD diffractometer
Absorption correction: multi-scan (*CrystalClear*; Rigaku, 2005 ▶) *T*_min_ = 0.958, *T*_max_ = 0.972
11123 measured reflections
5632 independent reflections
4018 reflections with *I* > 2σ(*I*)
*R*_int_ = 0.042

#### Refinement

*R*[*F* ^2^ > 2σ(*F* ^2^)] = 0.041
*wR*(*F* ^2^) = 0.107
*S* = 1.01
5632 reflections
340 parameters
H-atom parameters constrained
Δρ_max_ = 0.33 e Å^−3^
Δρ_min_ = −0.25 e Å^−3^

### 

Data collection: *CrystalClear* (Rigaku, 2005 ▶); cell refinement: *CrystalClear*; data reduction: *CrystalClear*; program(s) used to solve structure: *SHELXS97* (Sheldrick, 2008 ▶); program(s) used to refine structure: *SHELXL97* (Sheldrick, 2008 ▶); molecular graphics: *SHELXTL* (Sheldrick, 2008 ▶); software used to prepare material for publication: *SHELXTL*.

## Supplementary Material

Crystal structure: contains datablocks I, global. DOI: 10.1107/S1600536808041019/hb2872sup1.cif

Structure factors: contains datablocks I. DOI: 10.1107/S1600536808041019/hb2872Isup2.hkl

Additional supplementary materials:  crystallographic information; 3D view; checkCIF report

## Figures and Tables

**Fig. 1.**
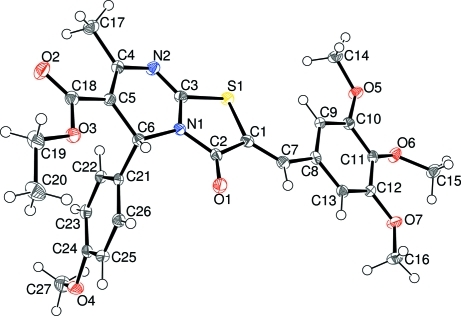
The molecular structure of (I), drawn with 50% probability ellipsoids (arbitrary spheres for the H atoms).

## supplementary crystallographic information

### Comment

Fused pyrimidine derivatives represent important target molecules due to their highly pronounced biological properties (Ashok *et al.*, 2007; Monks *et al.*, 1991). In this paper, the structure of the title compound, (I), is reported. The molecular structure of (I) is illustrated in Fig. 1.

The fused thiazole ring C1—C2—N1—C3—S1 has usual geometry as observed in other fused thiazolopyrimidine compounds (Liu *et al.*, 2004*a*,*b*; Sridhar *et al.*, 2006). The thiazole ring makes dihedral angles of 87.8 (2) and 17.9 (3)° with the benzene rings C21–C26 and C8–C13, respectively. The pyrimidine ring adopts a flattened boat conformation. The crystal packing of (I) is stabilized by π-π stacking interactions.

### Experimental

A mixture of ethyl 6-methyl-4-(4-methoxyphenyl)-2-thioxo-1,2,3,4- tetrahydropyrimidine-5-carboxylate (0.01 mol), chloroacetic acid (0.01 mol), fused sodium acetate (6 g) in glacial acetic acid (25 ml), acetic anhydride (10 ml) and 3,4,5-trimethoxybenzaldehyde (0.01 mol) was refluxed for 3 h. The reaction mixture was cooled and poured into cold water. The resulting solid was collected and crystallized from methanol to obtain the final product (80% yield, mp 448–449 K). ^1Ĥ NMR (CDCl~3~, p.p.m.): 1.23 (3*H*, m), 2.60 (3*H*, s), 3.78(3*H*,s),3.92(9*H*,m),4.13(2*H*,m),6.21 (1*H*, s), 6.73(2*H*,s), 6.85 (2*H*, d),7.36 (2*H*, d), 7.74 (1*H*,s).  The compound was recrystallized by slow evaporation of an ethanol solution, yielding yellow blocks of (I).

### Refinement

The H atoms were positioned geometrically with C—H = 0.93–0.98 Å and refined as riding with *U*_iso_ (H) = 1.2*U*_eq_(C) or 1.5*U*_eq_(methyl C).

### Crystal data

**Table:**

C_27_H_28_N_2_O_7_S	*Z* = 2
*M**_r_* = 524.57	*F*(000) = 552
Triclinic, *P*1	*D*_x_ = 1.394 Mg m^−^^3^
Hall symbol: -P 1	Melting point = 448–449 K
*a* = 10.485 (2) Å	Mo *K*α radiation, λ = 0.71073 Å
*b* = 10.854 (2) Å	Cell parameters from 3693 reflections
*c* = 11.318 (2) Å	θ = 1.9–27.5°
α = 83.42 (3)°	µ = 0.18 mm^−^^1^
β = 77.65 (3)°	*T* = 113 K
γ = 89.00 (3)°	Block, yellow
*V* = 1250.0 (4) Å^3^	0.24 × 0.18 × 0.16 mm

### Data collection

**Table:**

Rigaku Saturn CCD diffractometer	5632 independent reflections
Radiation source: rotating anode	4018 reflections with *I* > 2σ(*I*)
confocal	*R*_int_ = 0.042
Detector resolution: 7.31 pixels mm^-1^	θ_max_ = 27.5°, θ_min_ = 1.9°
ω and φ scans	*h* = −13→13
Absorption correction: multi-scan (*CrystalClear*; Rigaku, 2005)	*k* = −12→14
*T*_min_ = 0.958, *T*_max_ = 0.972	*l* = −13→14
11123 measured reflections	

### Refinement

**Table:**

Refinement on *F*^2^	Primary atom site location: structure-invariant direct methods
Least-squares matrix: full	Secondary atom site location: difference Fourier map
*R*[*F*^2^ > 2σ(*F*^2^)] = 0.041	Hydrogen site location: inferred from neighbouring sites
*wR*(*F*^2^) = 0.107	H-atom parameters constrained
*S* = 1.01	*w* = 1/[σ^2^(*F*_o_^2^) + (0.057*P*)^2^] where *P* = (*F*_o_^2^ + 2*F*_c_^2^)/3
5632 reflections	(Δ/σ)_max_ = 0.002
340 parameters	Δρ_max_ = 0.33 e Å^−^^3^
0 restraints	Δρ_min_ = −0.25 e Å^−^^3^

### Special details

**Table:**

Geometry. All e.s.d.'s (except the e.s.d. in the dihedral angle between two l.s. planes) are estimated using the full covariance matrix. The cell e.s.d.'s are taken into account individually in the estimation of e.s.d.'s in distances, angles and torsion angles; correlations between e.s.d.'s in cell parameters are only used when they are defined by crystal symmetry. An approximate (isotropic) treatment of cell e.s.d.'s is used for estimating e.s.d.'s involving l.s. planes.
Refinement. Refinement of *F*^2^ against ALL reflections. The weighted *R*-factor *wR* and goodness of fit *S* are based on *F*^2^, conventional *R*-factors *R* are based on *F*, with *F* set to zero for negative *F*^2^. The threshold expression of *F*^2^ > σ(*F*^2^) is used only for calculating *R*-factors(gt) *etc*. and is not relevant to the choice of reflections for refinement. *R*-factors based on *F*^2^ are statistically about twice as large as those based on *F*, and *R*- factors based on ALL data will be even larger.

### Fractional atomic coordinates and isotropic or equivalent isotropic displacement parameters (Å^2^)

**Table:**

	*x*	*y*	*z*	*U*_iso_*/*U*_eq_	
S1	1.07890 (3)	0.91459 (4)	0.32208 (3)	0.01833 (11)	
N1	0.86811 (11)	1.04443 (13)	0.32885 (11)	0.0150 (3)	
N2	1.04753 (12)	1.11583 (13)	0.17148 (12)	0.0199 (3)	
O1	0.72626 (10)	0.95600 (11)	0.49886 (9)	0.0221 (3)	
O2	0.79838 (11)	1.43070 (12)	0.09675 (10)	0.0278 (3)	
O3	0.64354 (10)	1.32735 (11)	0.23970 (10)	0.0205 (3)	
O4	0.42971 (11)	0.82312 (12)	0.11235 (10)	0.0255 (3)	
O5	1.35655 (9)	0.56820 (11)	0.49941 (10)	0.0214 (3)	
O6	1.24509 (10)	0.37222 (11)	0.62307 (10)	0.0225 (3)	
O7	0.98144 (10)	0.37091 (11)	0.73924 (10)	0.0225 (3)	
C1	0.94447 (13)	0.87479 (16)	0.44286 (13)	0.0166 (3)	
C2	0.83361 (14)	0.95891 (15)	0.43168 (13)	0.0154 (3)	
C3	0.99406 (13)	1.03930 (15)	0.26268 (13)	0.0161 (3)	
C4	0.97211 (14)	1.22004 (16)	0.14268 (13)	0.0177 (3)	
C5	0.84306 (14)	1.23011 (15)	0.19470 (13)	0.0156 (3)	
C6	0.77098 (13)	1.12589 (15)	0.28340 (13)	0.0148 (3)	
H6	0.7168	1.1634	0.3541	0.018*	
C7	0.92965 (14)	0.78128 (15)	0.53324 (13)	0.0174 (3)	
H7	0.8471	0.7793	0.5881	0.021*	
C8	1.01696 (14)	0.68271 (15)	0.56224 (13)	0.0161 (3)	
C9	1.15188 (13)	0.68154 (15)	0.51196 (13)	0.0156 (3)	
H9	1.1927	0.7507	0.4592	0.019*	
C10	1.22455 (13)	0.57908 (15)	0.53991 (13)	0.0163 (3)	
C11	1.16642 (14)	0.47343 (15)	0.61507 (13)	0.0158 (3)	
C12	1.03251 (14)	0.47620 (16)	0.66753 (13)	0.0165 (3)	
C13	0.96062 (14)	0.58019 (15)	0.64275 (13)	0.0166 (3)	
H13	0.8708	0.5825	0.6811	0.020*	
C14	1.42504 (14)	0.67531 (16)	0.43143 (15)	0.0223 (4)	
H14A	1.3988	0.6921	0.3531	0.033*	
H14B	1.5192	0.6603	0.4172	0.033*	
H14C	1.4040	0.7469	0.4774	0.033*	
C15	1.23394 (15)	0.29536 (17)	0.73613 (15)	0.0261 (4)	
H15A	1.2244	0.3476	0.8026	0.039*	
H15B	1.3125	0.2447	0.7339	0.039*	
H15C	1.1572	0.2411	0.7495	0.039*	
C16	0.84708 (14)	0.37320 (17)	0.80105 (15)	0.0232 (4)	
H16A	0.8338	0.4411	0.8525	0.035*	
H16B	0.8239	0.2942	0.8519	0.035*	
H16C	0.7918	0.3859	0.7411	0.035*	
C17	1.05115 (15)	1.31680 (18)	0.05259 (15)	0.0262 (4)	
H17A	1.0379	1.3080	−0.0292	0.039*	
H17B	1.1439	1.3061	0.0539	0.039*	
H17C	1.0233	1.3995	0.0742	0.039*	
C18	0.76540 (14)	1.34015 (16)	0.16950 (13)	0.0182 (3)	
C19	0.55395 (15)	1.42883 (17)	0.22755 (17)	0.0269 (4)	
H19A	0.5610	1.4598	0.1406	0.032*	
H19B	0.5742	1.4980	0.2706	0.032*	
C20	0.41855 (16)	1.37926 (19)	0.28279 (17)	0.0324 (5)	
H20A	0.3995	1.3115	0.2389	0.049*	
H20B	0.3550	1.4457	0.2769	0.049*	
H20C	0.4131	1.3483	0.3686	0.049*	
C21	0.68099 (14)	1.05058 (15)	0.23002 (13)	0.0150 (3)	
C22	0.72323 (14)	1.00178 (15)	0.12062 (13)	0.0168 (3)	
H22	0.8091	1.0202	0.0749	0.020*	
C23	0.64275 (15)	0.92636 (16)	0.07619 (13)	0.0187 (3)	
H23	0.6736	0.8927	0.0015	0.022*	
C24	0.51625 (15)	0.90098 (16)	0.14285 (14)	0.0188 (3)	
C25	0.47053 (14)	0.95390 (16)	0.25009 (14)	0.0193 (4)	
H25	0.3829	0.9397	0.2933	0.023*	
C26	0.55228 (14)	1.02701 (15)	0.29392 (13)	0.0174 (3)	
H26	0.5210	1.0616	0.3680	0.021*	
C27	0.47355 (17)	0.76215 (18)	0.00619 (15)	0.0294 (4)	
H27A	0.4963	0.8241	−0.0652	0.044*	
H27B	0.4039	0.7080	−0.0042	0.044*	
H27C	0.5506	0.7124	0.0149	0.044*	

### Atomic displacement parameters (Å^2^)

**Table:**

	*U*^11^	*U*^22^	*U*^33^	*U*^12^	*U*^13^	*U*^23^
S1	0.01300 (17)	0.0183 (2)	0.0222 (2)	0.00274 (15)	−0.00159 (14)	−0.00044 (16)
N1	0.0122 (5)	0.0157 (7)	0.0170 (6)	0.0011 (5)	−0.0034 (5)	−0.0009 (5)
N2	0.0158 (6)	0.0206 (8)	0.0208 (7)	0.0009 (6)	−0.0002 (5)	0.0008 (6)
O1	0.0149 (5)	0.0287 (7)	0.0198 (5)	0.0038 (5)	−0.0003 (4)	0.0025 (5)
O2	0.0297 (6)	0.0207 (7)	0.0289 (6)	0.0022 (5)	−0.0024 (5)	0.0063 (5)
O3	0.0165 (5)	0.0171 (6)	0.0270 (6)	0.0034 (5)	−0.0043 (4)	0.0000 (5)
O4	0.0257 (6)	0.0267 (7)	0.0262 (6)	−0.0093 (5)	−0.0089 (5)	−0.0039 (5)
O5	0.0133 (5)	0.0206 (7)	0.0264 (6)	0.0021 (5)	0.0009 (4)	0.0030 (5)
O6	0.0215 (5)	0.0194 (7)	0.0229 (6)	0.0066 (5)	−0.0012 (4)	0.0050 (5)
O7	0.0157 (5)	0.0193 (7)	0.0285 (6)	−0.0007 (5)	0.0001 (4)	0.0053 (5)
C1	0.0141 (6)	0.0186 (9)	0.0176 (7)	0.0004 (6)	−0.0033 (6)	−0.0041 (6)
C2	0.0151 (6)	0.0155 (8)	0.0167 (7)	0.0015 (6)	−0.0060 (6)	−0.0019 (6)
C3	0.0121 (6)	0.0164 (9)	0.0197 (8)	0.0020 (6)	−0.0025 (6)	−0.0034 (6)
C4	0.0173 (7)	0.0192 (9)	0.0162 (7)	−0.0004 (7)	−0.0040 (6)	0.0003 (6)
C5	0.0175 (7)	0.0148 (8)	0.0148 (7)	−0.0012 (6)	−0.0042 (6)	−0.0014 (6)
C6	0.0134 (6)	0.0143 (8)	0.0163 (7)	0.0017 (6)	−0.0023 (5)	−0.0018 (6)
C7	0.0143 (6)	0.0188 (9)	0.0193 (8)	0.0023 (6)	−0.0035 (6)	−0.0030 (7)
C8	0.0175 (7)	0.0175 (9)	0.0146 (7)	0.0019 (6)	−0.0061 (6)	−0.0028 (6)
C9	0.0153 (6)	0.0173 (9)	0.0146 (7)	−0.0013 (6)	−0.0047 (6)	0.0002 (6)
C10	0.0144 (6)	0.0188 (9)	0.0157 (7)	0.0019 (6)	−0.0028 (5)	−0.0032 (6)
C11	0.0170 (7)	0.0151 (9)	0.0157 (7)	0.0036 (6)	−0.0053 (6)	−0.0008 (6)
C12	0.0170 (7)	0.0165 (9)	0.0154 (7)	−0.0017 (6)	−0.0034 (6)	0.0013 (6)
C13	0.0137 (6)	0.0197 (9)	0.0165 (7)	0.0020 (6)	−0.0037 (5)	−0.0017 (6)
C14	0.0159 (7)	0.0235 (10)	0.0244 (8)	−0.0023 (7)	0.0011 (6)	0.0006 (7)
C15	0.0203 (7)	0.0244 (10)	0.0296 (9)	0.0017 (7)	−0.0034 (7)	0.0098 (8)
C16	0.0170 (7)	0.0243 (10)	0.0247 (8)	−0.0025 (7)	0.0024 (6)	−0.0001 (7)
C17	0.0196 (7)	0.0283 (11)	0.0267 (9)	−0.0032 (7)	−0.0004 (7)	0.0056 (8)
C18	0.0206 (7)	0.0186 (9)	0.0166 (7)	−0.0002 (7)	−0.0057 (6)	−0.0035 (7)
C19	0.0229 (8)	0.0212 (10)	0.0385 (10)	0.0092 (7)	−0.0102 (7)	−0.0057 (8)
C20	0.0203 (8)	0.0355 (12)	0.0430 (11)	0.0075 (8)	−0.0080 (7)	−0.0092 (9)
C21	0.0152 (6)	0.0131 (8)	0.0169 (7)	0.0005 (6)	−0.0064 (6)	0.0027 (6)
C22	0.0164 (6)	0.0158 (9)	0.0176 (7)	0.0004 (6)	−0.0043 (6)	0.0011 (6)
C23	0.0231 (7)	0.0183 (9)	0.0155 (7)	0.0022 (7)	−0.0060 (6)	−0.0017 (6)
C24	0.0203 (7)	0.0159 (9)	0.0219 (8)	−0.0018 (7)	−0.0109 (6)	0.0034 (7)
C25	0.0158 (7)	0.0199 (9)	0.0218 (8)	−0.0022 (7)	−0.0047 (6)	0.0012 (7)
C26	0.0169 (7)	0.0181 (9)	0.0169 (7)	0.0012 (6)	−0.0034 (6)	−0.0010 (6)
C27	0.0357 (9)	0.0259 (11)	0.0307 (10)	−0.0048 (8)	−0.0135 (8)	−0.0076 (8)

### Geometric parameters (Å, °)

**Table:**

S1—C3	1.7552 (18)	C11—C12	1.4039 (19)
S1—C1	1.7616 (15)	C12—C13	1.382 (2)
N1—C3	1.3754 (17)	C13—H13	0.9500
N1—C2	1.390 (2)	C14—H14A	0.9800
N1—C6	1.474 (2)	C14—H14B	0.9800
N2—C3	1.281 (2)	C14—H14C	0.9800
N2—C4	1.416 (2)	C15—H15A	0.9800
O1—C2	1.2147 (17)	C15—H15B	0.9800
O2—C18	1.212 (2)	C15—H15C	0.9800
O3—C18	1.3526 (17)	C16—H16A	0.9800
O3—C19	1.449 (2)	C16—H16B	0.9800
O4—C24	1.3727 (19)	C16—H16C	0.9800
O4—C27	1.424 (2)	C17—H17A	0.9800
O5—C10	1.3693 (17)	C17—H17B	0.9800
O5—C14	1.431 (2)	C17—H17C	0.9800
O6—C11	1.3686 (19)	C19—C20	1.505 (2)
O6—C15	1.429 (2)	C19—H19A	0.9900
O7—C12	1.369 (2)	C19—H19B	0.9900
O7—C16	1.4338 (16)	C20—H20A	0.9800
C1—C7	1.342 (2)	C20—H20B	0.9800
C1—C2	1.483 (2)	C20—H20C	0.9800
C4—C5	1.3632 (19)	C21—C22	1.385 (2)
C4—C17	1.501 (2)	C21—C26	1.4006 (19)
C5—C18	1.469 (2)	C22—C23	1.391 (2)
C5—C6	1.518 (2)	C22—H22	0.9500
C6—C21	1.519 (2)	C23—C24	1.393 (2)
C6—H6	1.0000	C23—H23	0.9500
C7—C8	1.451 (2)	C24—C25	1.391 (2)
C7—H7	0.9500	C25—C26	1.381 (2)
C8—C13	1.406 (2)	C25—H25	0.9500
C8—C9	1.4078 (19)	C26—H26	0.9500
C9—C10	1.382 (2)	C27—H27A	0.9800
C9—H9	0.9500	C27—H27B	0.9800
C10—C11	1.410 (2)	C27—H27C	0.9800
			
C3—S1—C1	91.65 (8)	O6—C15—H15A	109.5
C3—N1—C2	116.73 (14)	O6—C15—H15B	109.5
C3—N1—C6	120.95 (12)	H15A—C15—H15B	109.5
C2—N1—C6	121.87 (11)	O6—C15—H15C	109.5
C3—N2—C4	116.26 (12)	H15A—C15—H15C	109.5
C18—O3—C19	117.21 (13)	H15B—C15—H15C	109.5
C24—O4—C27	117.49 (12)	O7—C16—H16A	109.5
C10—O5—C14	116.92 (13)	O7—C16—H16B	109.5
C11—O6—C15	120.08 (12)	H16A—C16—H16B	109.5
C12—O7—C16	117.54 (13)	O7—C16—H16C	109.5
C7—C1—C2	119.69 (13)	H16A—C16—H16C	109.5
C7—C1—S1	129.97 (13)	H16B—C16—H16C	109.5
C2—C1—S1	110.26 (11)	C4—C17—H17A	109.5
O1—C2—N1	122.85 (15)	C4—C17—H17B	109.5
O1—C2—C1	127.13 (15)	H17A—C17—H17B	109.5
N1—C2—C1	110.02 (12)	C4—C17—H17C	109.5
N2—C3—N1	126.15 (15)	H17A—C17—H17C	109.5
N2—C3—S1	122.60 (11)	H17B—C17—H17C	109.5
N1—C3—S1	111.22 (11)	O2—C18—O3	122.02 (15)
C5—C4—N2	122.62 (14)	O2—C18—C5	128.08 (14)
C5—C4—C17	124.96 (16)	O3—C18—C5	109.89 (13)
N2—C4—C17	112.40 (13)	O3—C19—C20	107.07 (15)
C4—C5—C18	123.03 (14)	O3—C19—H19A	110.3
C4—C5—C6	121.00 (15)	C20—C19—H19A	110.3
C18—C5—C6	115.96 (12)	O3—C19—H19B	110.3
N1—C6—C5	108.39 (12)	C20—C19—H19B	110.3
N1—C6—C21	110.15 (13)	H19A—C19—H19B	108.6
C5—C6—C21	114.06 (12)	C19—C20—H20A	109.5
N1—C6—H6	108.0	C19—C20—H20B	109.5
C5—C6—H6	108.0	H20A—C20—H20B	109.5
C21—C6—H6	108.0	C19—C20—H20C	109.5
C1—C7—C8	131.59 (13)	H20A—C20—H20C	109.5
C1—C7—H7	114.2	H20B—C20—H20C	109.5
C8—C7—H7	114.2	C22—C21—C26	118.61 (14)
C13—C8—C9	118.62 (15)	C22—C21—C6	121.88 (12)
C13—C8—C7	116.98 (13)	C26—C21—C6	119.49 (13)
C9—C8—C7	124.36 (14)	C21—C22—C23	121.50 (13)
C10—C9—C8	119.55 (14)	C21—C22—H22	119.2
C10—C9—H9	120.2	C23—C22—H22	119.2
C8—C9—H9	120.2	C22—C23—C24	119.01 (14)
O5—C10—C9	125.01 (14)	C22—C23—H23	120.5
O5—C10—C11	113.31 (14)	C24—C23—H23	120.5
C9—C10—C11	121.69 (13)	O4—C24—C25	115.17 (13)
O6—C11—C12	125.18 (14)	O4—C24—C23	124.71 (14)
O6—C11—C10	116.08 (12)	C25—C24—C23	120.10 (15)
C12—C11—C10	118.54 (15)	C26—C25—C24	120.12 (13)
O7—C12—C13	124.32 (13)	C26—C25—H25	119.9
O7—C12—C11	115.93 (15)	C24—C25—H25	119.9
C13—C12—C11	119.73 (14)	C25—C26—C21	120.55 (14)
C12—C13—C8	121.71 (13)	C25—C26—H26	119.7
C12—C13—H13	119.1	C21—C26—H26	119.7
C8—C13—H13	119.1	O4—C27—H27A	109.5
O5—C14—H14A	109.5	O4—C27—H27B	109.5
O5—C14—H14B	109.5	H27A—C27—H27B	109.5
H14A—C14—H14B	109.5	O4—C27—H27C	109.5
O5—C14—H14C	109.5	H27A—C27—H27C	109.5
H14A—C14—H14C	109.5	H27B—C27—H27C	109.5
H14B—C14—H14C	109.5		
			
C3—S1—C1—C7	178.36 (16)	C8—C9—C10—C11	1.9 (2)
C3—S1—C1—C2	1.65 (12)	C15—O6—C11—C12	−42.5 (2)
C3—N1—C2—O1	178.34 (15)	C15—O6—C11—C10	142.70 (15)
C6—N1—C2—O1	−9.4 (2)	O5—C10—C11—O6	−8.2 (2)
C3—N1—C2—C1	−2.69 (19)	C9—C10—C11—O6	171.73 (14)
C6—N1—C2—C1	169.60 (13)	O5—C10—C11—C12	176.62 (13)
C7—C1—C2—O1	2.0 (3)	C9—C10—C11—C12	−3.5 (2)
S1—C1—C2—O1	179.09 (14)	C16—O7—C12—C13	−5.8 (2)
C7—C1—C2—N1	−176.92 (14)	C16—O7—C12—C11	175.85 (13)
S1—C1—C2—N1	0.18 (16)	O6—C11—C12—O7	5.1 (2)
C4—N2—C3—N1	5.0 (2)	C10—C11—C12—O7	179.79 (13)
C4—N2—C3—S1	−172.73 (11)	O6—C11—C12—C13	−173.40 (14)
C2—N1—C3—N2	−173.96 (15)	C10—C11—C12—C13	1.3 (2)
C6—N1—C3—N2	13.7 (2)	O7—C12—C13—C8	−175.95 (14)
C2—N1—C3—S1	3.99 (17)	C11—C12—C13—C8	2.4 (2)
C6—N1—C3—S1	−168.38 (11)	C9—C8—C13—C12	−4.0 (2)
C1—S1—C3—N2	174.90 (14)	C7—C8—C13—C12	173.80 (14)
C1—S1—C3—N1	−3.13 (12)	C19—O3—C18—O2	−2.0 (2)
C3—N2—C4—C5	−10.3 (2)	C19—O3—C18—C5	179.21 (13)
C3—N2—C4—C17	168.19 (14)	C4—C5—C18—O2	5.3 (3)
N2—C4—C5—C18	177.23 (14)	C6—C5—C18—O2	−174.66 (16)
C17—C4—C5—C18	−1.1 (3)	C4—C5—C18—O3	−175.97 (14)
N2—C4—C5—C6	−2.8 (2)	C6—C5—C18—O3	4.04 (19)
C17—C4—C5—C6	178.91 (14)	C18—O3—C19—C20	163.14 (14)
C3—N1—C6—C5	−23.72 (19)	N1—C6—C21—C22	−71.70 (18)
C2—N1—C6—C5	164.31 (13)	C5—C6—C21—C22	50.4 (2)
C3—N1—C6—C21	101.71 (16)	N1—C6—C21—C26	106.86 (16)
C2—N1—C6—C21	−70.26 (17)	C5—C6—C21—C26	−131.01 (15)
C4—C5—C6—N1	18.5 (2)	C26—C21—C22—C23	−2.7 (2)
C18—C5—C6—N1	−161.48 (13)	C6—C21—C22—C23	175.85 (15)
C4—C5—C6—C21	−104.57 (17)	C21—C22—C23—C24	0.9 (2)
C18—C5—C6—C21	75.42 (17)	C27—O4—C24—C25	−177.30 (15)
C2—C1—C7—C8	176.39 (16)	C27—O4—C24—C23	1.3 (2)
S1—C1—C7—C8	−0.1 (3)	C22—C23—C24—O4	−176.48 (15)
C1—C7—C8—C13	−162.98 (17)	C22—C23—C24—C25	2.1 (2)
C1—C7—C8—C9	14.6 (3)	O4—C24—C25—C26	175.59 (15)
C13—C8—C9—C10	1.8 (2)	C23—C24—C25—C26	−3.1 (3)
C7—C8—C9—C10	−175.79 (15)	C24—C25—C26—C21	1.2 (3)
C14—O5—C10—C9	5.4 (2)	C22—C21—C26—C25	1.7 (2)
C14—O5—C10—C11	−174.66 (13)	C6—C21—C26—C25	−176.93 (15)
C8—C9—C10—O5	−178.22 (14)		
